# Enhanced extracellular production of raw starch-degrading α-amylase in *Bacillus subtilis* through expression regulatory element modification and fermentation optimization

**DOI:** 10.1186/s12934-023-02116-z

**Published:** 2023-06-29

**Authors:** Dongbang Yao, Xudong Han, Huanhuan Gao, Bin Wang, Zemin Fang, He Li, Wei Fang, Yazhong Xiao

**Affiliations:** 1 School of Life Sciences, Anhui University, Hefei, 230601 China; 2 Anhui Key Laboratory of Modern Biomanufacturing, Hefei, 230601 China; 3 Anhui Provincial Engineering Technology Research Center of Microorganisms and Biocatalysis, Hefei, 230601 China; 4 AHU Green Industry Innovation Research Institute, Hefei, 230088 China

**Keywords:** Raw starch-degrading α-amylase, *Bacillus subtilis*, Promoter, Signal peptide, RBS sequence, Fermentation optimization

## Abstract

**Background:**

Raw starch-degrading α-amylase (RSDA) can hydrolyze raw starch at moderate temperatures, thus contributing to savings in starch processing costs. However, the low production level of RSDA limits its industrial application. Therefore, improving the extracellular expression of RSDA in *Bacillus subtilis*, a commonly used industrial expression host, has great value.

**Results:**

In this study, the extracellular production level of *Pontibacillus* sp. ZY raw starch-degrading α-amylase (AmyZ1) in *B. subtilis* was enhanced by expression regulatory element modification and fermentation optimization. As an important regulatory element of gene expression, the promoter, signal peptide, and ribosome binding site (RBS) sequences upstream of the *amyZ1* gene were sequentially optimized. Initially, based on five single promoters, the dual-promoter P_*veg*_-P_*ylB*_ was constructed by tandem promoter engineering. Afterward, the optimal signal peptide SP_NucB_ was obtained by screening 173 *B. subtilis* signal peptides. Then, the RBS sequence was optimized using the RBS Calculator to obtain the optimal RBS1. The resulting recombinant strain WBZ-VY-B-R1 showed an extracellular AmyZ1 activity of 4824.2 and 41251.3 U/mL during shake-flask cultivation and 3-L fermenter fermentation, which were 2.6- and 2.5-fold greater than those of the original strain WBZ-Y, respectively. Finally, the extracellular AmyZ1 activity of WBZ-VY-B-R1 was increased to 5733.5 U/mL in shake flask by optimizing the type and concentration of carbon source, nitrogen source, and metal ions in the fermentation medium. On this basis, its extracellular AmyZ1 activity was increased to 49082.1 U/mL in 3-L fermenter by optimizing the basic medium components as well as the ratio of carbon and nitrogen sources in the feed solution. This is the highest production level reported to date for recombinant RSDA production.

**Conclusions:**

This study represents a report on the extracellular production of AmyZ1 using *B. subtilis* as a host strain, and achieved the current highest expression level. The results of this study will lay a foundation for the industrial application of RSDA. In addition, the strategies employed here also provide a promising way for improving other protein production in *B. subtilis*.

**Supplementary Information:**

The online version contains supplementary material available at 10.1186/s12934-023-02116-z.

## Background

Starch, as an important and abundant carbohydrate in nature, can be used in food, bioethanol, papermaking, pharmaceutical, textile, and other industries. In starch processing, α-amylase is often used to hydrolyze the internal α-1,4 glycosidic bonds and degrade them into low-polymerization dextrins as well as maltose and glucose [1]. Traditional starch processing requires thermostable α-amylase, but there are problems such as high energy consumption and complicated process due to its action temperature of 95–105 ℃ [2]. On the contrary, using raw starch-degrading α-amylases (RSDA) can effectively solve the above problems, because it can efficiently hydrolyze raw starch below the gelatinization temperature (40 °C), so receiving increased attention. However, the low production level of RSAD is one of the important factors limiting its practical application in industry. Therefore, achieving efficient production of RSDA has great industrial prospects and beneficial economic benefits.

*Bacillus subtilis* is an attractive expression host for achieving high expression of RSDA for several reasons. *B. subtilis* is a Gram-positive bacterium, that has been widely used as a cell factory for the production of various industrial enzymes due to its generally regarded as safe (GRAS) nature, excellent secretory capability, low nutritional demands, and well-established fermentation processes. Although many heterologous proteins have achieved high expression in *B. subtilis*, such as pullulanase [3], protease [4], keratinase [5], etc., the recombinant expression level of certain target proteins is still low due to sequence specificity. To overcome the above problems, various expression strategies including expression regulatory element modification and fermentation optimization have been developed to improve the expression level of target proteins [6]. Among them, the modified expression regulatory elements include promoter, signal peptide (SP), 5′-untranslated region (5′-UTR), terminator, etc.

Promoters are key gene expression regulatory elements that control the intensity and timing of gene expression, its engineering serves as an important strategy to increase target protein expression in *B. subtilis*. For example, based on the screening of nine strongly single promoters (P_*HpaII*_, P_*spoVG*_, P_*luxS*_, P_*amyE*_, P_*43*_, P_*gsiB*_, P_*srfA*_, P_*nprE*_, and P_*sigW*_), promoter P_*amyE*_ significantly increased the expression of D-allulose 3-epimerase (SfDAE) in *B. subtilis*. What’s more, the expression level of SfDAE was further increased by 91% by constructing dual-promoter P_*amyE*_-P_*HpaII*_ [7]. Similarly, Wu et al. reported that lipase A activity mediated by promoter P_*43*_ was approximately twice that of promoter P_*HpaII*_ by screening nine single promoters (P_*HpaII*_, P_*43*_, P_*amyE*_, P_*fusA*_, P_*hag*_, P_*sodA*_, P_*srfA*_, P_*tufA*_, and P_*ylb*_), and on this basis, the constructed dual-promoter P_*43*_-P_*hag*_ increased lipase A activity by 26% compared to single promoter P_*43*_ [8].

The signal peptide is a special amino acid sequence at the N-terminal of the protein precursor, which plays an important role in helping the protein precursors maintain a transportable unfolded state and coordinating the whole secretion process [9]. Since the specific relationship between signal peptide sequence and target protein production is still unclear, irrational or semi-rational screening is currently an important way to obtain the optimal signal peptide [10]. For example, based on high-throughput screening technology, Yao et al. randomly screened 173 *B. subtilis* signal peptides and obtained the optimal signal peptide SP_YojL_ which can increase α-amylase activity by 3.5 times compared with control SP_AmyQ’_ [11]. In another study, Guo et al. used a semi-rational screening method to obtain the optimal signal peptide SP_WapA_ from 13 *B. subtilis* signal peptides, which can increase the sucrose isomerase activity by more than 2-fold compared with control SP_WapA_ [12].

The ribosome binding site (RBS) is the core of 5’-UTR and regulates gene expression at the translational level by affecting translation initiation efficiency [13]. Optimization of RBS sequences is a common and effective strategy to improve the yield of recombinant proteins in *B. subtilis*. For example, Fang et al. improved the keratinase activity by 69% through the prediction of RBS translation efficiency and multi-site saturation mutation screening [5]. To efficiently obtain optimal RBS sequences, Salis et al. developed an RBS Calculator to assist the optimization design of RBS, which can improve the target translation initiation rate in *Escherichia coli* by 100,000 times [14]. In a previous study, Niu et al. performed RBS sequence optimization based on the RBS Calculator to increase type I L-asparaginase (BlAase) activity by 1.39-fold [15].

In addition to the performance of the production strain itself, the fermentation culture conditions are also an important factor affecting its production level [16]. As important nutrients, carbon and nitrogen sources in culture medium have a significant influence on strain fermentation and functional biological product production [17]. Therefore, the current research on fermentation optimization usually focuses on optimizing the carbon and nitrogen sources of the fermentation medium, based on which target protein expression level in *B. subtilis* is also effectively improved. For example, Zhu et al. improved the recombinant collagenase activity in *B. subtilis* by 95% by optimizing the carbon source of the culture medium [18]. In addition, Zhang et al. optimized the composition of carbon and nitrogen sources in the feed solution, thereby increasing the extracellular activity of recombinant pullulanase in *B. subtilis* by 2.4-fold [19].

**Fig. 1:**
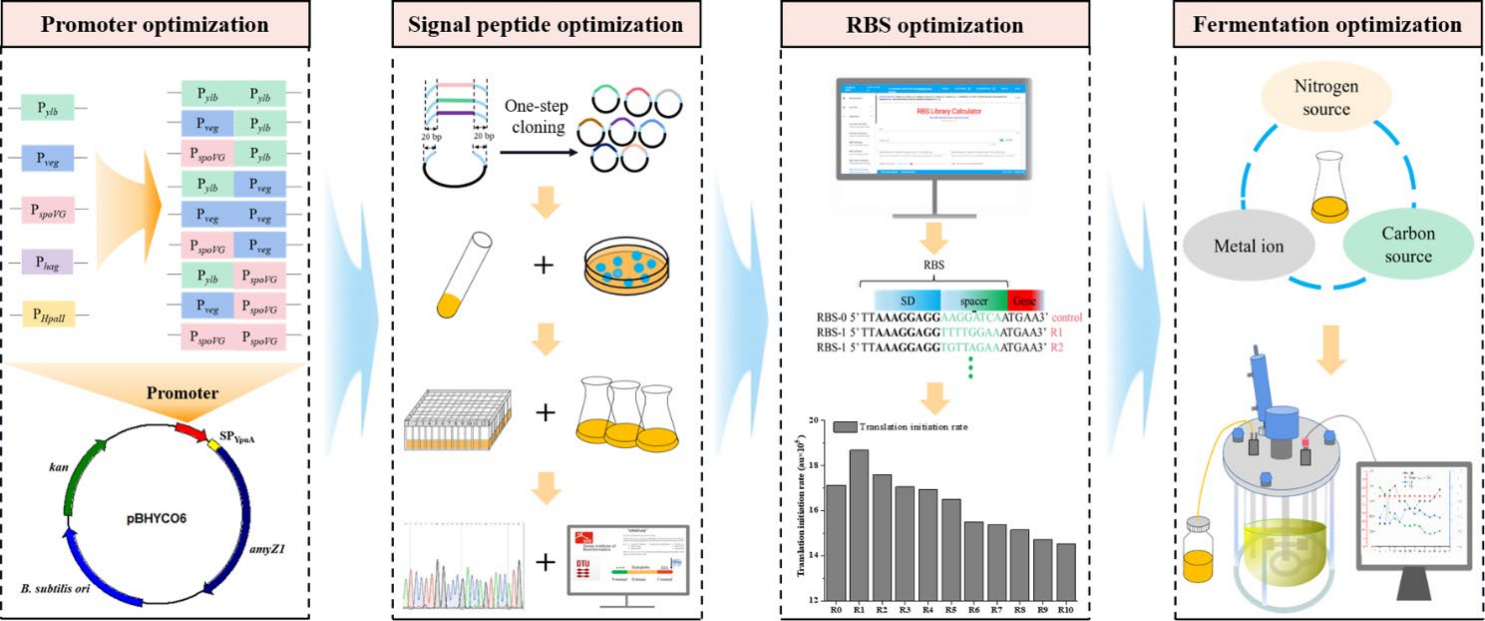
Schematic diagram of the expression strategy used to enhance AmyZ1 production

In a previous study, we obtained a novel RSDA (AmyZ1) with promising industrial applications from the marine bacterium *Pontibacillus* sp. ZY [20]. However, the recombinant production of AmyZ1 in available *B. subtilis* strains has been relatively poor so far [21]. Therefore, the objective of this study was to improve the extracellular expression level of AmyZ1 in *B. subtilis*. In initial experiments, the promoter elements are optimized based on single promoter screening and tandem promoter construction. Then, the signal peptide elements were optimized based on irrational random screening 173 *B. subtilis* signal peptides. Next, the RBS sequence within the 5’-UTR was optimized using the RBS Calculator. Finally, the extracellular production capacity of the constructed *B. subtilis* recombinant strain was confirmed in a 3-L fermenter, and the extracellular AmyZ1 activity was further improved by optimizing the components of the fermentation medium and feed solution. A schematic representation of the expression strategy adopted in this study is shown in Fig. 1.

## Results

### Effect of promoter element on AmyZ1 expression

To investigate the effect of promoter element on the extracellular AmyZ1 expression in *B. subtilis*, five constitutive promoters (P_*spoVG*_, P_*veg*_, P_*ylb*_, P_*HpaII*_, and P_*hag*_), which were reported to exhibit strong transcript levels in *B. subtilis* [13, 22–24], were screened. The nucleotide sequences of these promoters are shown in Additional file 1: Table S1. Based on these promoters, the corresponding AmyZ1 recombinant vectors were transferred into *B. subtilis* WB600 to obtain strains WBZ-S (P_*spoVG*_), WBZ-V (P_*veg*_), WBZ-Y (P_*ylb*_), WBZ-H (P_*HpaII*_), and WBZ-G (P_*hag*_), respectively (Table 1).

**Table 1:** Strains and plasmids used in this study

Strains or plasmids	Properties	References
Strains		
*E. coli* JM109	Clone strain	Takara
*Pontibacillus sp*. ZY	Clone strain	Our laboratory
*B. subtilis* 168	*trpC2*, clone strain	Our laboratory
*B. subtilis* WB600	*B. subtilis* 168 derivate, deficient in *nprE*, *aprE*, *epr*, *bpr*, *mpr*, *nprB*, expression strain	Our laboratory
WBZ-S	*B. subtilis* WB600/pBHZ-S	This work
WBZ-V	*B. subtilis* WB600/pBHZ-V	This work
WBZ-Y	*B. subtilis* WB600/pBHZ-Y	This work
WBZ-H	*B. subtilis* WB600/pBHZ-H	This work
WBZ-G	*B. subtilis* WB600/pBHZ-G	This work
WBZ-YY	*B. subtilis* WB600/pBHZ-YY	This work
WBZ-VY	*B. subtilis* WB600/pBHZ-VY	This work
WBZ-SY	*B. subtilis* WB600/pBHZ-SY	This work
WBZ-YV	*B. subtilis* WB600/pBHZ-YV	This work
WBZ-VV	*B. subtilis* WB600/pBHZ-VV	This work
WBZ-SV	*B. subtilis* WB600/pBHZ-SV	This work
WBZ-YS	*B. subtilis*/pBHZ-YS	This work
WBZ-VS	*B. subtilis* WB600/pBHZ-VS	*This work*
WBZ-SS	*B. subtilis* WB600/pBHZ-SS	This work
WBZ-VY-B	*B. subtilis* strain c, WBZ-VY derivative, SP_NucB_	This work
*B. subtilis* strain a	WBZ-VY derivative, SP_LipB_	This work
*B. subtilis* strain b	WBZ-VY derivative, SP_NprB_	This work
*B. subtilis* strain d	WBZ-VY derivative, SP_YjcN_	This work
*B. subtilis* strain e	WBZ-VY derivative, SP_Pbp_	This work
*B. subtilis* strain f	WBZ-VY derivative, SP_Ydbk_	This work
*B. subtilis* strain g	WBZ-VY derivative, SP_Pel_	This work
*B. subtilis* strain h	WBZ-VY derivative, SP_YndA_	This work
*B. subtilis* strain i	WBZ-VY derivative, SP_Mpr_	This work
WBZ-VY-B-Rn	*B. subtilis* WB600/pBHZ-VY-B-Rn, n represents 1–10	This work
Plasmids		
pBHYCO6	pBHSSC1 derivative, *amyZ1* gene,P_*spoVG*_-P_*spoVG142*_, SP_YpuA_ optimized sequence O6	[21]
pBHZ-S	pBHYCO6 derivative, replace the original promoter with P_*spoVG*_	This work
pBHZ-V	pBHYCO6 derivative, replace the original promoter with P_*veg*_	This work
pBHZ-Y	pBHYCO6 derivative, replace the original promoter with P_*ylB*_	This work
pBHZ-H	pBHYCO6 derivative, replace the original promoter with P_*HpaII*_	This work
pBHZ-G	pBHYCO6 derivative, replace the original promoter with P_*hag*_	This work
pBHZ-YY	pBHYCO6 derivative, replace the original promoter with P_*ylB*_-P_*ylB*_	This work
pBHZ-VY	pBHYCO6 derivative, replace the original promoter with P_*veg*_-P_*ylB*_	This work
pBHZ-SY	pBHYCO6 derivative, replace the original promoter with P_*spoVG*_-P_*ylB*_	This work
pBHZ-YV	pBHYCO6 derivative, replace the original promoter with P_*ylB*_-P_*veg*_	This work
pBHZ-VV	pBHYCO6 derivative, replace the original promoter with P_*veg*_-P_*veg*_	This work
pBHZ-SV	pBHYCO6 derivative, replace the original promoter with P_*spoVG*_-P_*veg*_	This work
pBHZ-YS	pBHYCO6 derivative, replace the original promoter with P_*ylB*_-P_*spoVG*_	This work
pBHZ-VS	pBHYCO6 derivative, replace the original promoter with P_*veg*_-P_*ylB*_	This work
pBHZ-SS	pBHYCO6 derivative, replace the original promoter with P_*spoVG*_-P_*spoVG*_	This work
pBHZ-VY-SP_x_	pBHZ-VY derivative, replace the original signal peptide with SP_x_, SP_x_ represents any one of the 173 Sec-type signal peptides	This work
pBHZ-VY-B	pBHZ-VY derivative, replace the original signal peptide with SP_NucB_	This work
pBHZ-VY-B-Rn	pBHZ-VY-B derivative, replace the original RBS with RBSn, n represents 1–10	This work

**Fig. 2:**
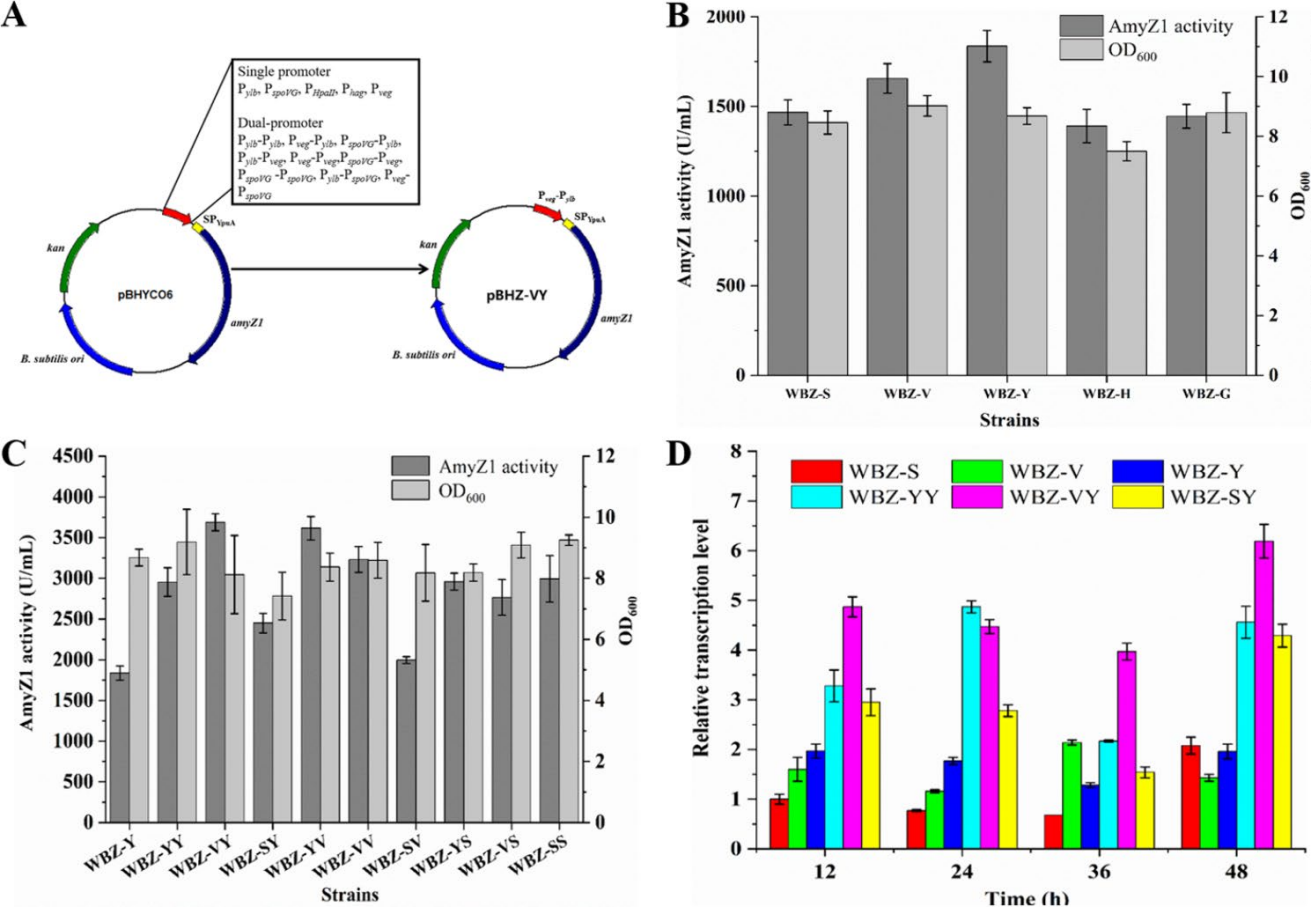
Effect of promoter optimization on the extracellular AmyZ1 production. (A) Schematic diagram of expression vectors construction with various promoters. (B) Effect of single promoters on extracellular AmyZ1 production. (C) Effect of dual-promoters on extracellular AmyZ1 production. (D) Relative transcription levels of *amyZ1* in *B. subtilis* strains. Error bars represent the standard deviation

After shake-flask culture (Fig. 2B), the highest extracellular AmyZ1 activity was produced by WBZ-Y (1836.2 U/mL) containing P_*ylb*_, followed by WBZ-V (1655.9 U/mL) containing P_*veg*_, and WBZ-S (1466.9 U/mL) containing P_*spoVG*_. The extracellular AmyZ1 activity of WBZ-Y is 1.32-fold higher than that of WBZ-H (1390.8 U/mL), which contained P_*HpaII*_ and had the lowest extracellular activity.

To further enhance the expression level of AmyZ1, the identified superior promoters P_*ylb*_, P_*veg*_, and P_*spoVG*_ were used to construct the dual promoter plasmids (Fig. 2A), and obtain corresponding AmyZ1 production strains WBZ-YY (P_*ylb*_-P_*ylb*_), WBZ-VY (P_*veg*_-P_*ylb*_), WBZ-SY (P_*spoVG*_-P_*ylb*_), WBZ-YV (P_*ylb*_-P_*veg*_), WBZ-VV (P_*veg*_-P_*veg*_), WBZ-SV (P_*spoVG*_-P_*veg*_), WBZ-YS (P_*ylb*_-P_*spoVG*_), WBZ-VS (P_*veg*_-P_*spoVG*_), and WBZ-SS (P_*spoVG*_-P_*spoVG*_), respectively (Table 1). As shown in Fig. 2C, the extracellular AmyZ1 activity of these strains was higher than that of WBZ-Y containing the best single promoter P_*ylb*_, especially the AmyZ1 activity of WBZ-VY (3687.7 U/mL) containing the double promoter P_*veg*_-P_*ylb*_ was 2.0-fold higher than that of WBZ-Y (1836.2 U/mL). These results have been verified by SDS-PAGE analysis (Additional file 1: Fig. S1).

To investigate whether the extracellular AmyZ1 activity of recombinant strains containing different promoters is related to their promoter transcription intensity, quantitative real-time PCR (qRT-PCR) was used to detect the mRNA levels of *amyZ1* in these recombinant strains. As shown in Fig. 2D, the transcriptional intensity of the P_*veg*_-P_*ylb*_ was consistently higher than that of any other dual-promoter combination (except for 24 h) and single promoter throughout the fermentation process, which was consistent with the AmyZ1 activity (Fig. 2C). Since the strain WBZ-VY had the highest extracellular AmyZ1 activity, the corresponding P_*veg*_-P_*ylb*_ was selected for subsequent studies.

### Effect of signal peptide element on AmyZ1 expression

**Fig. 3:**
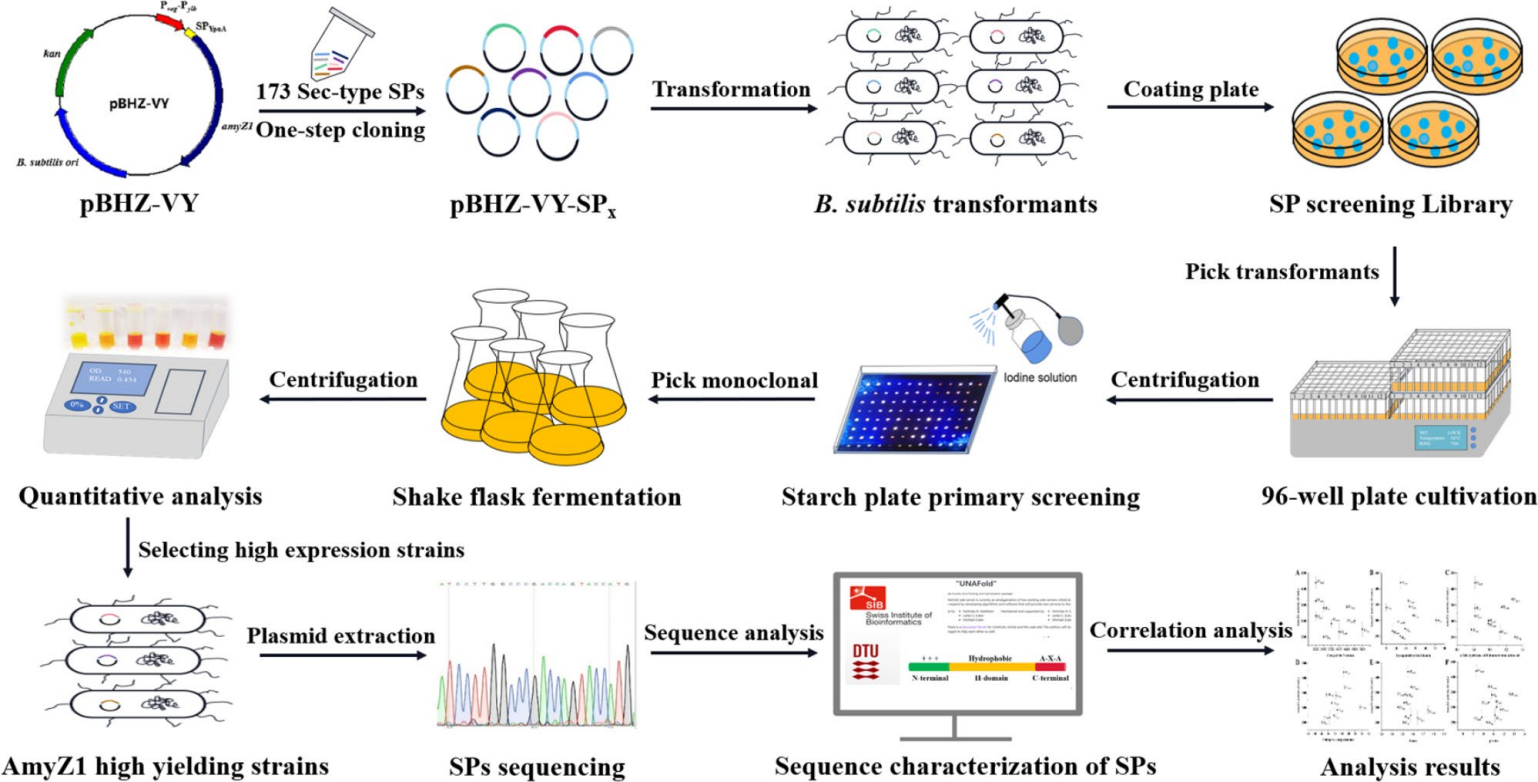
Workflow of signal peptide library construction, high-throughput screening, and sequence analysis

**Fig. 4:**
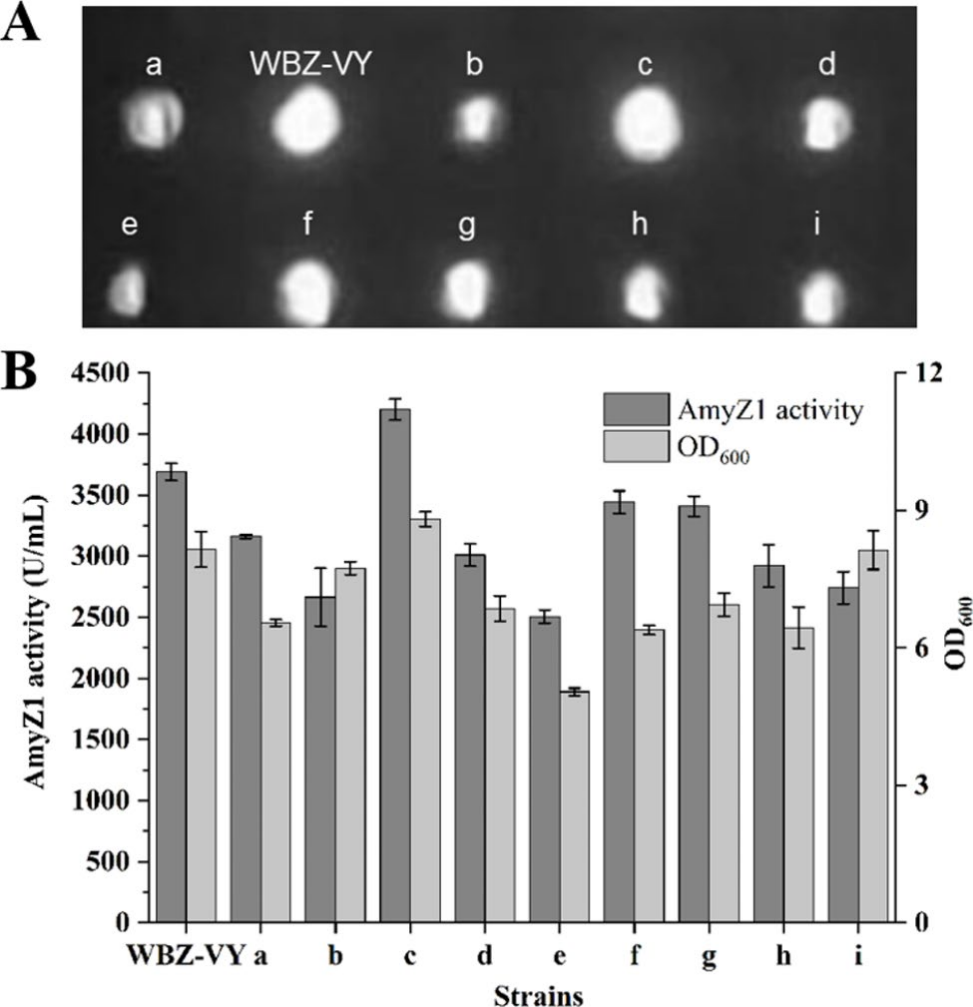
Effect of signal peptide optimization on the extracellular AmyZ1 production. (A) Results of starch plate screening of signaling peptide library. (B) Extracellular AmyZ1 activity of *B. subtilis* strains in shake flask fermentation. Error bars represent the standard deviation

To obtain an efficient signal peptide in coordination with the dual-promoter P_*veg*_-P_*ylb*_, a signal peptide screening library containing 173 *B. subtilis* Sec-type signal peptides was constructed and high-throughput screening was performed (Fig. 3). More than 1730 clones (10-fold over-sampling) were obtained from 19 96-well plates, but only one clone (named c) had a larger transparent circle compared to the control strain WBZ-VY (Fig. 4A). Then, the clone c was selected and fermented in a triangular shake flask to verify its ability to express AmyZ1. After shake-flask culture (Fig. 4B), the extracellular AmyZ1 activity of strain c (4199.1 U/mL) was 1.14-fold (p < 0.05) greater than that of control strain WBZ-VY (3687.7 U/mL). Sequencing analysis revealed that the signal peptide in strain c was SP_NucB_ (Additional file 2: Table S1). For the convenience of subsequent studies, we renamed strain c as WBZ-VY-B (Table 1).

To further enhance the extracellular expression level of AmyZ1 by rationally modifying the signal peptide SP_NucB_, we investigated the relationship between signal peptide sequence features and their mediated extracellular AmyZ1 activity. Therefore, we first randomly selected eight transformants (named a, b, d, e, f, g, h, and i, respectively) with different transparent circle sizes based on the qualitative assay results of AmyZ1 activity (Fig. 4A). The extracellular AmyZ1 activities of these eight clones were then verified by shake flask fermentation and found to be consistent with the size of their transparent circles in the starch plate (Fig. 4). Sequencing analysis revealed that the signal peptides in these strains were SP_LipB_, SP_NprB_, SP_YjcN_, SP_Pbp_, SP_Ydbk_, SP_Pel_, SP_YndA_, and SP_Mpr_, respectively (Additional file 2: Table S1).

**Fig. 5:**
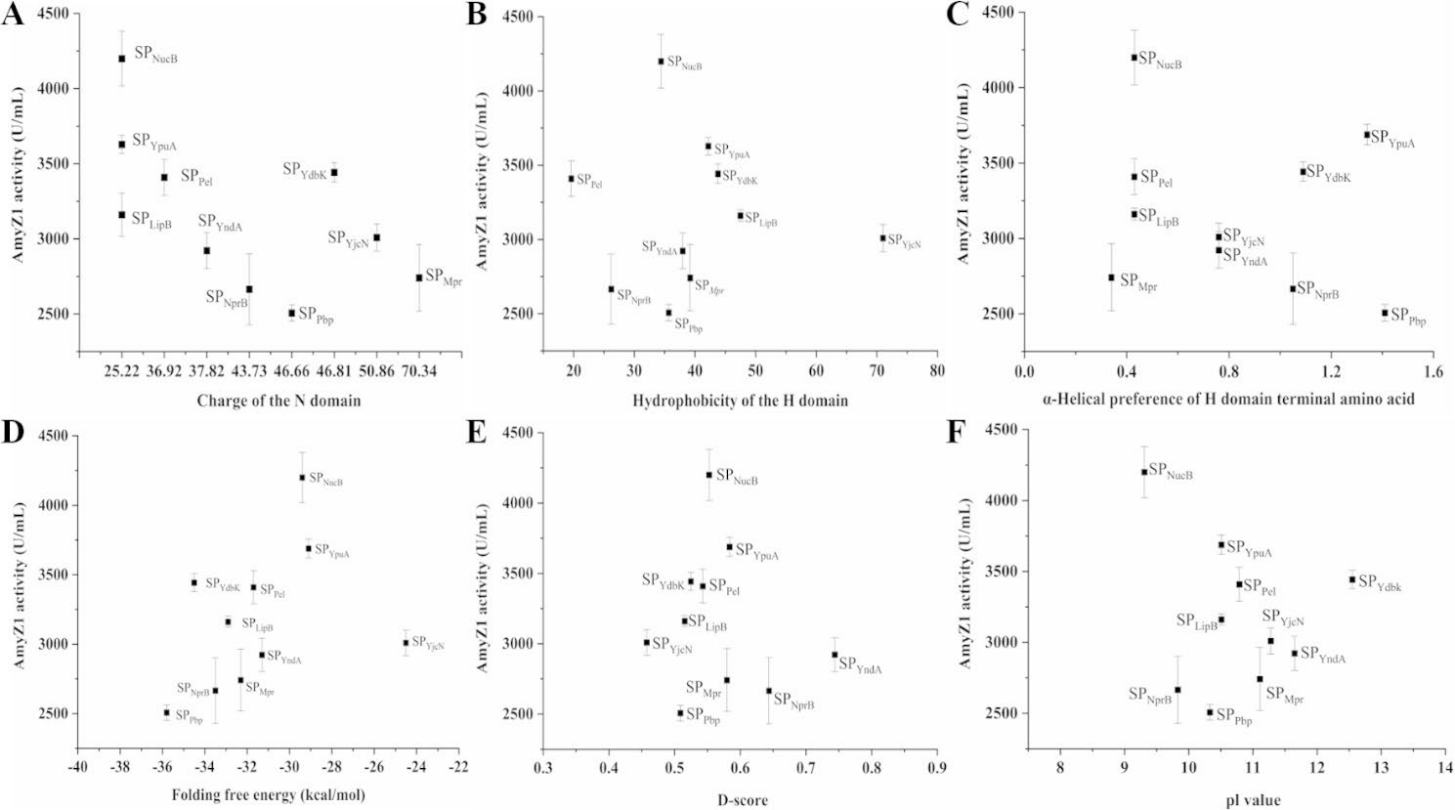
Effect of signal peptide sequence characteristics on extracellular AmyZ1 production. (A) Charge of N domain. (B) Hydrophobicity of the H domain. (C) α-Helix preference of H domain terminal amino acid. (D) Folding free energy. (E) D-score. (F) pI value. Error bars represent the standard deviation

We then analyzed the sequence properties of all the above signal peptides including the N domain charge, the H domain hydrophobicity, the H domain terminal amino acid α-helical preference, the folding free energy, the D-score, and the pI value (Additional file 2: Table S1). The correlation between the above sequence properties of signal peptides and their mediated extracellular AmyZ1 activity was then analyzed (Fig. 5). However, no significant linear relationship was found between them (Fig. 5). Since SP_NucB_ provided the greatest increase in extracellular AmyZ1 activity, WBZ-VY-B was selected for subsequent studies.

### Effect of RBS element on AmyZ1 expression

In this study, we optimized the RBS sequence in the *amyZ1* expression vector based on the RBS Calculator (https://salislab.net/software/, accessed on 16 November 2022) and selected the top 10 RBS optimized sequences (RBS1 to RBS10) from the 8000 sequences obtained according to the predicted translation initial rate (TIR) values (Additional file 1: Table S2).

**Fig. 6:**
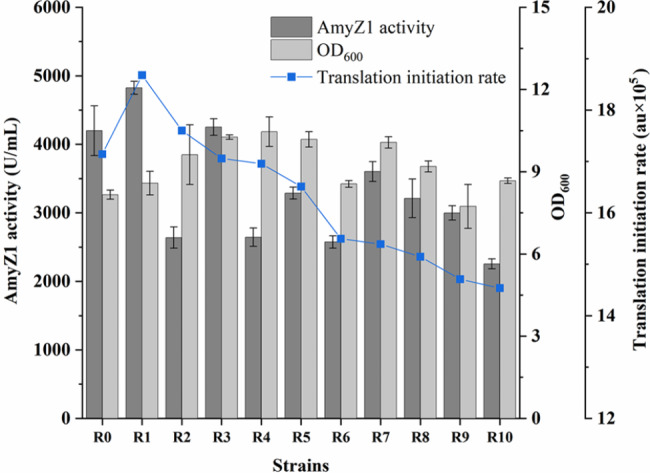
Effect of RBS sequence optimization on extracellular AmyZ1 production. Error bars represent the standard deviation

Then, the original RBS sequence (RBS0) was replaced using the optimized RBS (RBS1 to RBS10) to construct the corresponding recombinant strains WBZ-VY-B-R1 to WBZ-VY-B-R10, respectively (Table 1). As shown in Fig. 6, after shake-flask culture, the extracellular AmyZ1 activity of WBZ-VY-B-R1 (4824.2 U/mL) containing RBS1 was the highest, 1.15-fold (p < 0.05) higher than that of the control strain WBZ-VY-B (4199.1 U/mL) containing RBS0. Although the TIR of RBS2 was also higher than that of RBS0 (Additional file 1: Table S2), the extracellular AmyZ1 activity of WBZ-VY-B-R2 (2639.1 U/mL) was only 62.8% of that of WBZ-VY-B (Fig. 6). In addition, the extracellular AmyZ1 activity of WBZ-VY-B-R7 (3603.4 U/mL) was 1.37-fold greater than that of WBZ-VY-B-R2, while the TIR of RBS7 was lower than that of RBS2. Therefore, there was no significant linear relationship between the TIR of the RBS sequence and its extracellular AmyZ1 activity (Fig. 6).

### Scale-up of AmyZ1 production in a 3-L fermenter

**Fig. 7:**
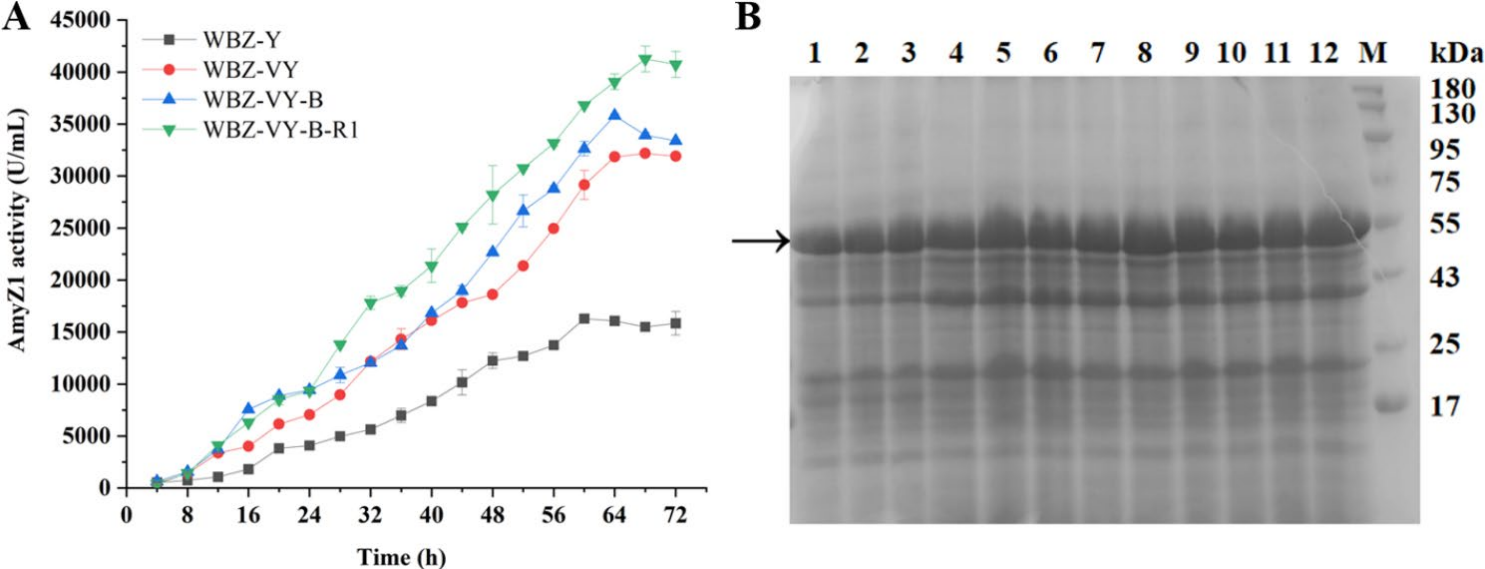
Scale-up (3-L) fermentation of *B. subtilis* strains. (A) The AmyZ1 activity in the fermentation supernatant was monitored as a function of time. Error bars represent the standard deviation. (B) SDS-PAGE analysis of *B. subtilis* strains fermentation supernatant. The arrow indicates the band corresponding to AmyZ1 (~ 55 kDa). Lanes 1–3: supernatant samples of WBZ-Y at 56, 60, and 64 h, respectively. Lanes 4–6: supernatant samples of WBZ-VY at 64, 68, and 72 h, respectively. Lanes 7–9: supernatant samples of WBZ-VY-B at 60, 64, and 68 h, respectively. Lanes 10–12: supernatant samples of WBZ-VY-B-R1 at 64, 68, and 72 h, respectively. Lane M: protein molecular weight markers

To further verify the effect of expression regulatory element optimization on the extracellular expression of AmyZ1 in *B. subtilis*, the recombinant strains WBZ-Y, WBZ-VY, WBZ-VY-B, and WBZ-VY-B-R1 were fermented in a 3-L fermenter, respectively. The extracellular AmyZ1 activities of WBZ-VY (32179.5 U/mL), WBZ-VY-B (35824.4 U/mL), and WBZ-VY-B-R1 (41251.3 U/mL) after 3-L fermenter fermentation were 2.0-, 2.2-, and 2.5-fold higher than that of WBZ-Y (16280.8 U/mL), respectively (Fig. 7A). These results are consistent with those of the shake flask fermentation and have been verified by SDS-PAGE analysis (Fig. 7B).

### Enhancing AmyZ1 production through fermentation optimization

Since WBZ-VY-B-R1 exhibited great potential for the industrial production of AmyZ1, to obtain higher extracellular AmyZ1 activity, the fermentation medium components were first optimized at the shake flask fermentation level. Then, based on this, the basic medium and feed solution of 3-L fermenter fermentation were further optimized.

**Fig. 8:**
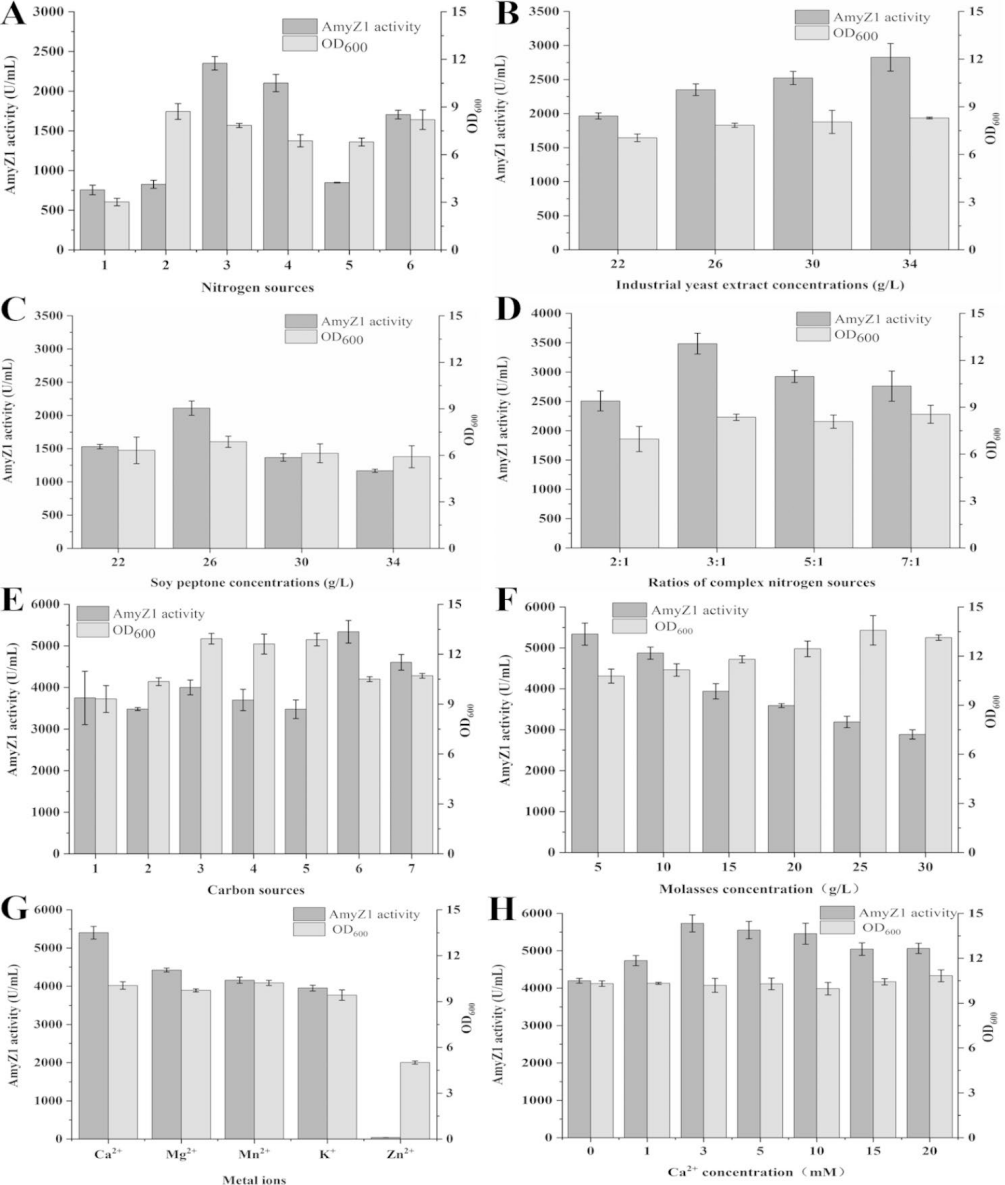
Optimization of shake flask fermentation medium components. (A) Effect of different nitrogen sources. 1, ammonium chloride; 2, soybean meal powder; 3, industrial yeast extract; 4, soy peptone; 5, bran; 6, tryptone. (B) Effect of varying the industrial yeast extract concentration. (C) Effect of varying the soy peptone concentration. (D) Effect of different ratios of complex nitrogen sources. (E) Effect of different carbon sources. 1, glucose; 2, glycerol; 3, soluble starch; 4, cassava starch; 5, corn starch; 6, molasses; 7, sucrose. (F) Effect of varying the molasses concentration. (G) Effect of different metal ions. (H) Effect of varying the calcium ion concentration. Error bars represent the standard deviation

In initial experiments, six nitrogen sources including ammonium chloride, soybean meal powder, industrial yeast extract, soy peptone, bran, and tryptone were used to replace peptone and yeast extract in the original shake flask fermentation medium, respectively, at 26 g/L. As shown in Fig. 8A, the best single nitrogen source for AmyZ1 production by WBZ-VY-B-R1 was industrial yeast extract, followed by soy peptone. Moreover, the optimal industrial yeast extract concentration was found to be 34 g/L (Fig. 8B), while the optimal soybean peptone concentration remained at 26 g/L (Fig. 8C).

Considering the price and AmyZ1 activity yield, a composite nitrogen source containing industrial yeast extract and soy peptone was selected for subsequent experiments. The effect of the ratio between industrial yeast extract and soy peptone on the AmyZ1 production by WBZ-VY-B-R1 was investigated while the total concentration was kept at 34 g/L. It was found that the highest extracellular AmyZ1 activity of 3484.6 U/mL was achieved when the ratio was 3:1 (Fig. 8D).

Then, different carbon sources (5 g/L of glucose, glycerol, soluble starch, cassava starch, corn starch, molasses, or sucrose) were selected for the modified fermentation medium. Compared with other carbon sources, molasses was most suitable for WBZ-VY-B-R1 to produce AmyZ1 (Fig. 8E). What’s more, the extracellular AmyZ1 activity of WBZ-VY-B-R1 reached a maximum (5338.5 U/mL) when the molasses concentration was 5 g/L (Fig. 8F).

Next, different metal ions (10 mM of Ca^2+^, Mg^2+^, Mn^2+^, K^+^, and Zn^2+^) were selected for the modified fermentation medium. As shown in Fig. 8G, the highest AmyZ1 activity was obtained in the presence of Ca^2+^. In addition, the results of Ca^2+^ concentration optimization showed that the highest AmyZ1 activity (5733.5 U/mL) was obtained when its concentration was 3 mM (Fig. 8H).

Based on the above shake flask fermentation optimization results, the basic medium for 3-L fermenter fermentation was modified to 5 g/L molasses, 25.5 g/L industrial yeast extract, 8.5 g/L soybean peptone, 3 mM Ca^2+^, 7.5 g/L NaCl, and 3 mL/L trace element solution. The feed solution for 3-L fermenter fermentation was modified to 400 g/L molasses, 60 g/L industrial yeast extract, 20 g/L soy peptone, 3 mM Ca^2+^, and 30 mL/L trace element solution.

**Fig. 9:**
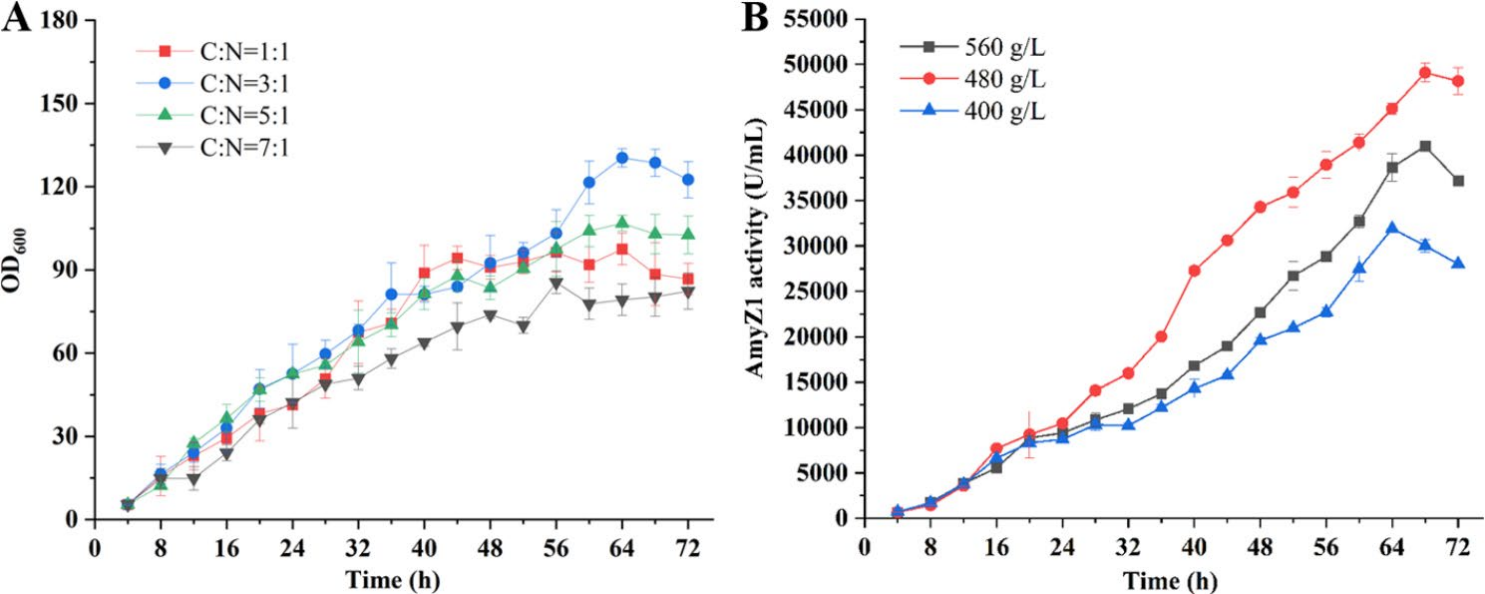
Optimization of feed solution components. (A) Optimization of the ratio of carbon and nitrogen sources. (B) Optimization of the total concentration of carbon and nitrogen sources. Error bars represent the standard deviation

Then, the ratio of carbon and nitrogen sources in the feed solution was investigated. Their ratios (w/w) were set to 1:1, 3:1, 5:1, and 7:1, respectively, while their total concentrations were kept at 480 g/L. As shown in Fig. 9A, the carbon and nitrogen sources ratio of 3:1 achieved the highest AmyZ1 activity. The result has been verified by SDS-PAGE analysis (Additional file 1: Fig. S2A).

Next, the total concentration (400, 480, or 560 g/L) of carbon and nitrogen sources in feed solution was investigated according to the principle of a 3:1 ratio of carbon to nitrogen sources. As shown in Fig. 9B, the highest AmyZ1 activity of 49082.1 U/mL was achieved at a total concentration of 480 g/L. The SDS-PAGE results of the WBZ-VY-B-R1 3-L fermenter fermentation supernatant at a total concentration of 480 g/L are shown in Additional file 1: Fig. S2B.

## Discussion

To improve the recombinant extracellular expression level of AmyZ1 in *B. subtilis*, a series of optimization studies on expression regulatory elements and recombinant strain fermentation were conducted.

Among the five promoters (P_*spoVG*_, P_*veg*_, P_*ylb*_, P_*HpaII*_, and P_*hag*_) used in this study, promoter P_*ylb*_ mediated the highest extracellular AmyZ1 activity, followed by P_*veg*_, P_*spoVG*_, P_*hag*_, and P_*HpaII*_ (Fig. 2B). There was no significant difference in extracellular AmyZ1 activity mediated by promoter P_*spoVG*_ and P_*hag*_ (P > 0.05). Promoters P_*spoVG*_, P_*veg*_, P_*ylb*_, and P_*hag*_ are endogenous promoters of *B. subtilis*, while P_*HpaII*_ is derived from *Staphylococcus aureus*. Thus, the extracellular AmyZ1 activity mediated by promoters P_*spoVG*_, P_*veg*_, P_*ylb*_, and P_*hag*_ higher than that of promoter P_*HpaII*_ may be due to the differences between species.

Promoters P_*spoVG*_, P_*veg*_, P_*ylb*_, and P_*hag*_ belong to σ^H^, σ^A^, σ^A^, and σ^D^, respectively [25]. Among them, *B. subtilis* is relatively rich in σ^A^. Therefore, it seems reasonable to speculate that the extracellular AmyZ1 activity mediated by promoters P_*ylb*_ and P_*veg*_ is higher than that of promoters P_*spoVG*_ and P_*hag*_ may be related to the type and amount of σ on which they depend. As shown in Fig. 2D, the transcriptional intensity of the P_*ylb*_ was consistently higher than that of P_*veg*_ (except for 36 h) throughout the fermentation process. This may be the reason why the extracellular AmyZ1 activity mediated by promoter P_*ylb*_ is higher than that of promoter P_*veg*_.

A dual promoter system consisting of two single promoters can effectively increase the expression level of target proteins by extending the transcriptional expression period or enhancing the transcriptional intensity during the expression period [26, 27]. In this study, the extracellular AmyZ1 activities mediated by the constructed dual promoter systems were higher than that of the best single promoter P_*ylb*_ (Fig. 2C). Our results of qRT-PCR for different promoter systems indicate that the tandem dual-promoter structure enhances target protein expression by increasing the transcriptional intensity. These results are consistent with previous studies [28, 29].

Although it is still impossible to predict in advance which signal peptide is optimal based on the target protein sequence, previous studies have shown that for a specific target protein, signal peptides with higher N domain charge, stronger H domain hydrophobicity, and stronger amino acid α-helix preference of H domain terminal amino acid are generally easier to recognize membrane transport channels and transmembrane transport, resulting in higher secretion efficiency [8, 11]. In addition, Fu et al. reported that among these signal peptide sequence parameters of D-score, pI value and folding free energy, there was good linear relationship between folding free energy and extracellular α-amylase activity (R^2^ = 0.76), since it affects translation initiation efficiency [30]. However, our signal peptide analysis results (Fig. 5) are inconsistent with the previously described data [11, 30].

Similar to the results of Lu et al. [31], and Fu et al. [30], there was no significant relationship between the D-score (Fig. 5E) and pI value (Fig. 5F) of signal peptide and its extracellular AmyZ1 activity. Based on the above results, it seems reasonable to speculate that the effect of the signal peptide on the extracellular target protein activity may be achieved through the combined action of its various sequence features.

The RBS1 obtained based on the RBS calculator increased the extracellular AmyZ1 activity by 15%, but it was also found that there was no significant linear relationship between the predicted TIR of the RBS sequence and its extracellular AmyZ1 activity (Fig. 6). In a previous study, Niu et al. reported similar results [15]. Since transcription, translation, secretion, folding, and their coordination all affect the final expression levels of target proteins, we speculated that the above differences might be caused by post-translational secretion or folding [32].

As shown in Fig. 8E, molasses was more suitable for the AmyZ1 production by WBZ-VY-B-R1 than glucose. Previous studies have shown that although fast-acting carbon sources facilitate rapid uptake and utilization by the bacterium, late-acting carbon sources can effectively inhibit the catabolic inhibition effect within the bacterium, so sometimes the production strain based on late-acting carbon sources exhibit higher production activity instead [17]. A similar result was reported by Li et al. [21]. In their study, glycerol was more favorable than glucose for the AmyZ1 production by *B. subtilis* [21].

The extracellular AmyZ1 activity of WBZ-VY-B-R1 with added Ca^2+^ is highest compared to other metal ions (Fig. 8G). Previous studies have shown that Ca^2+^ plays an important role in establishing a Ca^2+^-Na^+^-Ca^2+^ connection within the domain B of α-amylase and stabilizing the structure of the catalytic cleft, so Ca^2+^ can generally enhance the activity and stability of α-amylase [20, 33]. Therefore, it is reasonable to speculate that the addition of Ca^2+^ to increase the extracellular AmyZ1 activity may be due to its enhanced stability and catalytic activity of AmyZ1.

Fed-batch fermentation is a commonly used fermentation strategy because it is beneficial to cell growth and target protein production while optimizing the composition of carbon and nitrogen sources in feed solution can further improve the production level [19]. In this study, the extracellular AmyZ1 activity of WBZ-VY-B-R1 was 3.7-fold greater when the ratio of carbon and nitrogen sources in feed solution was 3:1 (49082.1 U/mL) than that when it was 7:1 (13252.6 U/mL). Combined with the growth curve of WBZ-VY-B-R1 (Additional file 1: Fig. S3), it is easy to find that the effect of different carbon and nitrogen source ratios on the extracellular AmyZ1 activity of WBZ-VY-B-R1 is achieved by affecting its cell growth vitality and cell density.

The highest level of recombinant RSDA production in *B. subtilis* in the previous study was reported by Li et al. [21]. In their study, *B. subtilis* BZYACO6 was cultured in a 3-L fermenter for 64 h and obtained an extracellular AmyZ1 activity of 25,070 U/mL. In this study, culturing WBZ-VY-B-R1 in a 3-L fermenter for 68 h produced an extracellular AmyZ1 activity (49082.1 U/mL) approximately 1.96-fold higher than that obtained with BZYACO6. Although this represents the highest expression level of RSDA so far, it may be further enhanced by optimizing the entire secretion process of AmyZ1 in *B. subtilis* [9]. In addition, the fermentation optimization of WBZ-VY-B-R1 in this study was carried out based on the principle of examining one variable at a time, but this neglected the combinatorial effect between variables. Therefore, subsequent screening of important influencing factors and studying their interactions based on a statistical analysis of response surface methodology will also contribute to further improving the expression level of AmyZ1 in *B. subtilis*.

## Conclusion

High-level expression of AmyZ1 in *B. subtilis* was achieved by expression regulatory element modification and fermentation optimization. By optimizing expression regulatory elements including promoter, signal peptide, and RBS sequences, the extracellular AmyZ1 activity of the obtained recombinant strain WBZ-VY-B-R1 was increased by 2.6- and 2.5-fold in shake flask cultivation and 3-L fermenter fermentation, respectively. After optimization of the fermentation medium components, *B. subtilis* WBZ-VY-B-R1 produced an extracellular AmyZ1 activity of 5733.5 U/mL in shake flask and 49082.1 U/mL in 3-L fermenter. This is the highest extracellular AmyZ1 activity achieved to date. Therefore, this study lays a foundation for the industrial production and application of AmyZ1. Moreover, the strategies employed here offer valuable guidance for improving other protein production in *B. subtilis*.

## Materials and methods

### Strains, plasmids, and media

All strains and plasmids used in this study are listed in Table 1. *E. coli* JM109 and *B. subtilis* WB600 were used as the host strain for plasmid construction and expression, respectively. Luria − Bertani (LB) medium was used for *E. coli* growth and seed fermentation of *B. subtilis*. The original fermentation medium used for shake flask fermentation is 2 × YT medium containing 10 mM calcium ions [2]. For 3-L fermenter fermentation, the original basic medium (w/v) contained 1% glycerol, 2.4% tryptone, 1.5% yeast extract, 0.75% NaCl, and 3 mL/L trace element solution [11]. The original feeding medium (w/v) contained 40% glycerol, 2% yeast extract, 6% tryptone, and 30 mL/L trace element solution.

### Plasmid construction

The primers used in this study are shown in Additional file 1: Table S3. The pBHZ fragment (the backbone of pBHZ-S) was obtained from pBHYCO6 [21] using primers F1/R1. The promoter P_*spoVG*_ was obtained from the *B. subtilis* WB600 genome using primers PS-F/PS-R. The plasmid pBHZ-S was created by linking the P_*spoVG*_ fragment with the pBHZ fragment using prolonged overlap extension polymerase chain reaction (POE-PCR). The P_*veg*_, P_*ylb*_, P_*HpaII*_, and P_*hag*_ fragments were obtained from the *B. subtilis* WB600 genome using primers PV-F/PV-R, PY-F/PY-R, PH-F/PH-R and PG-F/PG-R, respectively. The vectors pBHZ-V, pBHZ-Y, pBHZ-H, and pBHZ-G were obtained by replacing the P_*spoVG*_ fragment in the plasmid pBHZ-S with the P_*veg*_, P_*ylb*_, P_*HpaII*_, and P_*hag*_ fragments, respectively.

The pBHZ-Y fragment (the backbone of pBHZ-YY) was obtained from pBHZ-Y using primers F2/R2. The promoters P_*ylb*_, P_*veg*_, and P_*spoVG*_ were obtained from the *B. subtilis* WB600 genome using primers PYY-F/PYY-R, PVY-F/PVY-R and PGY-F/PGY-R, respectively. The plasmids pBHZ-YY, pBHZ-VY, and pBHZ-SY were obtained by linking the P_*ylb*_, P_*veg*_, and P_*spoVG*_ with the pBHZ-Y fragment using POE-PCR, respectively. The pBHZ-V fragment (the backbone of pBHZ-VV) was obtained from pBHZ-V using primers F3/R3. The promoters P_*ylb*_, P_*veg*_, and P_*spoVG*_ were obtained from the *B. subtilis* WB600 genome using primers PYV-F/PYV-R, PVV-F/PVV-R and PGV-F/PGV-R, respectively. The plasmids pBHZ-YV, pBHZ-VV, and pBHZ-SV were obtained by linking the P_*ylb*_, P_*veg*_, and P_*spoVG*_ with the pBHZ-V fragment using POE-PCR, respectively. The pBHZ-S fragment (the backbone of pBHZ-SS) was obtained from pBHZ-S using primers F4/R4. The promoters P_*ylb*_, P_*veg*_, and P_*spoVG*_ were obtained from the *B. subtilis* WB600 genome using primers PYS-F/PYS-R, PVS-F/PVS-R, and PGS-F/PGS-R, respectively. The plasmids pBHZ-YS, pBHZ-VS, and pBHZ-SS were obtained by linking the P_*ylb*_, P_*veg*,_ and P_*spoVG*_ with the pBHZ-S fragment using POE-PCR, respectively.

The pBHZ-VY fragment (the backbone of pBHZ-VY-SP_x_) was obtained from pBHZ-VY using primers F5/R5. The plasmid pBHZ-VY-SP_x_ was obtained by linking the pBHZ-VY fragment with 173 *B. subtilis* Sec-type signal peptides (Takara Bio Inc., Dalian, China) using ClonExpress II One Step Cloning Kit (Nazyme Bio Co., Nanjing, China), respectively.

The pBHZ-VY-B fragment (the backbone of pBHZ-VY-B-Rn, n represents 1–10 respectively) was obtained from pBHZ-VY-SP_NucB_ using primers F6/R6. The RBS1 to RBS10 were obtained from the pBHZ-VY-SP_NucB_ using primers R1-F/R1-R to R10-F/R10-R, respectively. The plasmids pBHZ-YV-B-R1 to pBHZ-YV-B-R10 were obtained by linking the RBS1 to RBS10 with the pBHZ-VY-B fragment using POE-PCR, respectively.

### Construction of signal peptide library and high-throughput screening

Construction of signal peptide library by transforming plasmid pBHZ-VY-SP_x_ into *B. subtilis* WB600. The signal peptide screening library is all positive transformants obtained after transformation of *B. subtilis* WB600 with the recombinant vector pBHZ-VY-SP_x_ (SP_x_ represents any one of the 173 Sec-type signal peptides). The obtained transformants were transferred into 96 deep-well plates containing 600 µL LB medium with 30 mg/L kanamycin and incubated at 37 °C and 800 rpm for 10 h. 50 µL the above-obtained culture solution was transferred to a new 96 deep-well plate containing 500 µL 2 × YT medium with 30 mg/L kanamycin and incubated at 37 °C and 800 rpm for 48 h. Then, after centrifugation (12,000 × *g*, 10 min), the fermentation supernatant was inoculated onto a starch screening plate (w/v) containing 2% soluble starch and 1% agar using a microplate replicator. The starch screening plate was placed at 30 ℃ for 2 h and then sprayed with iodine solution. Qualitative determination of AmyZ1 activity based on the size of the transparent circle produced on the starch plate. Finally, the transformant corresponding to the transparent circle with a larger diameter size was subjected to shake flask fermentation, thus further verifying its ability to produce AmyZ1.

### Shake flask and 3-L fermenter cultivation

The bacterial solution stored in the − 80 °C refrigerator was inoculated at 2% (v/v) into a 50-mL triangular flask containing 10 mL LB medium and incubated at 37 °C and 200 rpm for 8 h. The 1 mL above-obtained culture solution was then transferred to a 250-mL triangular flask containing 50 mL 2 × YT medium, and cultured at 37 °C and 200 rpm for 48 h. In the above shake flask fermentation, 30 mg/L kanamycin was added to LB and 2 × YT medium respectively.

Monoclonal activated by plate scribing was picked and inoculated into a 50-mL triangular flask containing 5 mL LB medium and incubated at 37 °C and 200 rpm for 10 h to obtain the primary seed solution. The primary seed solution was then transferred to a 250-mL triangular flask containing 100 mL LB medium at 2% (v/v) and cultured under the same conditions for 10 h to obtain the secondary seed solution. Then, 100 mL secondary seed solution was transferred to a 3-L fermenter containing 1.1 L basic fermentation medium and incubated at 37 ℃, pH 7.0, and 300 rpm. During the 3-L fermenter fermentation period, the dissolved oxygen in fermentation broth is maintained at 30% by coupling the stirring speed (300–800 rpm) and adjusting the proportion of oxygen in gas mixture. In addition, the pH of fermentation system was maintained at 7.0 by supplementing with NH_4_OH or 20% (v/v) HCl. When dissolved oxygen rebound occurred, the feed solution was supplemented at a flow rate of 16 mL/L·h. A certain volume of samples was collected at specific time intervals for subsequent AmyZ1 activity determination and sodium dodecyl sulfate–polyacrylamide gel electrophoresis (SDS‑PAGE) analysis. Both LB medium and basic fermentation medium were supplemented with 30 mg/L kanamycin, and the kanamycin was replenished once per 24 h during the 3-L fermenter fermentation process.

### Determination of cell concentration and AmyZ1 activity

The fermentation broth was appropriately diluted and its absorbance value at 600 nm was measured using an ultraviolet spectrophotometer (Shanghai optical instrument factory, Shanghai, China), which is the cell concentration OD_600_.

The AmyZ1 activity determination method was modified based on the report of Fang et al. [20]. Briefly, 0.3 mL of 2% (w/v) raw rice starch solution, prepared with NaH_2_PO_4_-Na_2_HPO_4_ buffer (50 mM, pH 7.0), was incubated at 40 ℃ for 10 min, and then 30 µL crude enzyme solution was added. The resulting solution was incubated for another 10 min, and 0.3 mL 3,5-dinitrosalicylic acid (DNS) was added and mixed. This mixture was incubated in boiling water for 15 min. Then, the reaction solution was immediately cooled in ice water and its absorbance value at 540 nm was measured using an ultraviolet spectrophotometer. The crude enzyme solution for AmyZ1 activity determination by this method is the supernatant obtained by centrifuging (12,000 × *g*, 10 min) the fermentation broth. One unit of AmyZ1 activity was defined as the amount of enzyme needed to release 1 µmol of reducing sugars as maltose per minute under standard assay conditions described above.

### SDS‑PAGE analysis

20 µL fermentation supernatant sample was taken, 20 µL 2 × loading buffer was added, then mixed using pipette blowing, and incubated in boiling water for 10 min. 8 µL the above solution was used for SDS-PAGE with 12% separation gel. After electrophoresis, the protein gel was submerged in staining solution (0.25% Coomassie Brilliant Blue R-250 solution) and heated to boiling in a microwave oven and maintained for 3 min. Then, the protein gel was rinsed with water and submerged in decolorization solution to decolorize protein bands until they were clear.

### Quantitative real‑time PCR

*B. subtilis* precipitates were collected from fermentation solution samples by centrifugation (12,000 × *g*, 10 min), and then the total RNA of the bacteria was extracted by the Bacteria Total RNA Isolation Kit (Sangon Biotech, Shanghai, China), and the corresponding cDNA was obtained by the Evo M-MLV RT Kit (AG, Hunan, China). The obtained cDNA was used as a template for qPCR, and the transcription level of *amyZ1* gene was determined with the 16 S rRNA gene as the reference gene. The primers for qPCR amplification of *amyZ1* gene and 16 S rRNA gene were F7/R7 and F8/R8 (Additional file 1: Table S3), respectively. qPCR was performed based on the Light-Cycler 96 Real-Time PCR system (Roche, Basel, Switzerland) using the SYBR Green Premix Pro Taq HS qPCR Kit (AG, Hunan, China). The qPCR amplification conditions included 95 ℃ for 30 s; followed by 35 cycles of 95 ℃ for 5 s, 60 ℃ for 30 s, and a melt-curve step (0.3 ℃/s, from 60 to 95 ℃). The data were analyzed using 2^−ΔΔCT^ methodology [34].

### Statistical analysis

All data in this report represent the mean (± standard deviation) of three independent experiments. Data were statistically analyzed using Student’s *t*-test and differences of P < 0.05 were considered statistically significant. Statistical analyses were performed using SAS statistical software (version 8.1, SAS Institute Inc., Cary, NC, USA).

## Electronic supplementary material

Below is the link to the electronic supplementary material.

**Additional file 1: Fig. S1** SDS-PAGE analysis of *B. subtilis* strains containing various promoters in shake flask fermentation. The arrow indicates the band corresponding to AmyZ1 (~55 kDa). Lanes 1-10: supernatant samples from WBZ-Y, WBZ-YY, WBZ-VY, WBZ-SY, WBZ-YV, WBZ-VV, WBZ-SV, WBZ-YS, WBZ-VS, and WBZ-SS, respectively. Lane M: protein molecular weight markers. **Fig. S2** SDS-PAGE analysis of WBZ-VY-B-R1 fermentation supernatant in a 3-L fermenter. (A) Lanes 1-3: supernatant samples at 64, 68, and 72 h at a 3:1 ratio, respectively. Lanes 4-6: supernatant samples at 64, 68, and 72 h at a 1:1 ratio, respectively. Lanes 7-9: supernatant samples at 60, 64, and 68 h at a 5:1 ratio, respectively. Lanes 10-12: supernatant samples at 52, 56, and 60 h at a 7:1 ratio, respectively. (B) Lanes 1-9: supernatant samples at 8, 24, 32, 40, 48, 56, 64, 68, and 72 h, respectively. The arrow indicates the band corresponding to AmyZ1 (~55 kDa). Lane M: protein molecular weight markers. **Fig. S3** Growth curve of WBZ-VY-B-R1 cultured in a 3-L fermenter. **Table S1** Nucleotide sequence of promoters. **Table S2** Optimized RBS sequence information. **Table S3** Primers used in this study.

**Additional file 2: Table S1** Sequence characteristics of signal peptides.

## Data Availability

All data generated or analyzed during this study are included in this published article and its additional files.

## Abbreviations

BlAase: type I L-asparaginase
DNS: 3,5-dinitrosalicylic acid
GRAS: generally recognized as safe
POE-PCR: prolonged overlap extension polymerase chain reaction
qRT-PCR: quantitative real-time PCR
RBS: ribosome binding site
RSDA: raw starch-degrading α-amylase
SDS‑PAGE: sodium dodecyl sulfate–polyacrylamide gel electrophoresis
SfDAE: D-allulose 3-epimerase
SP: signal peptide
TIR: translation initial rate
5′-UTR: 5′-untranslated region.
