# Supplementary material for: Enhanced extracellular production of raw starch-degrading α-amylase in Bacillus subtilis through expression regulatory element modification and fermentation optimization
Source: Microb Cell Fact. 2023 Jun 29;22:118. doi: 10.1186/s12934-023-02116-z (PMC10308679; doi:10.1186/s12934-023-02116-z)
Supplement: Supplementary file 1 — Additional file 1: Fig. S1 SDS-PAGE analysis of B. subtilis strains containing various promoters in shake flask fermentation. The arrow indicates the band corresponding to AmyZ1 (~55 kDa). Lanes 1-10: supernatant samples from WBZ-Y, WBZ-YY, WBZ-VY, WBZ-SY, WBZ-YV, WBZ-VV, WBZ-SV, WBZ-YS, WBZ-VS, and WBZ-SS, respectively. Lane M: protein molecular weight markers. Fig. S2 SDS-PAGE analysis of WBZ-VY-B-R1 fermentation supernatant in a 3-L fermenter. (A) Lanes 1-3: supernatant samples at 64, 68, and 72 h at a 3:1 ratio, respectively. Lanes 4-6: supernatant samples at 64, 68, and 72 h at a 1:1 ratio, respectively. Lanes 7-9: supernatant samples at 60, 64, and 68 h at a 5:1 ratio, respectively. Lanes 10-12: supernatant samples at 52, 56, and 60 h at a 7:1 ratio, respectively. (B) Lanes 1-9: supernatant samples at 8, 24, 32, 40, 48, 56, 64, 68, and 72 h, respectively. The arrow indicates the band corresponding to AmyZ1 (~55 kDa). Lane M: protein molecular weight markers. Fig. S3 Growth curve of WBZ-VY-B-R1 cultured in a 3-L fermenter. Table S1 Nucleotide sequence of promoters. Table S2 Optimized RBS sequence information. Table S3 Primers used in this study. [file 12934_2023_2116_MOESM1_ESM.docx]

**Additional information**

**Enhanced extracellular production of raw starch-degrading α-amylase in *Bacillus subtilis* through expression regulatory element modification and fermentation optimization**

Dongbang Yao^1,2,3,4†^, Xudong Han^1,2,3,4†^, Huanhuan Gao^1,2,3,4^, Bin Wang^1,2,3,4^, Zemin Fang^1,2,3,4^, He Li^1,2,3,4^, Wei Fang^1,2,3,4*^, Yazhong Xiao^1,2,3,4*^

^*^Correspondence: Wei Fang (fangahu@163.com); Yazhong Xiao (yzxiao@ahu.edu.cn)

^†^Dongbang Yao and Xudong Han contributed equally to this work

^1^ School of Life Sciences, Anhui University, Hefei, 230601, China.

^2^ Anhui Key Laboratory of Modern Biomanufacturing, Hefei, 230601, China.

^3^ Anhui Provincial Engineering Technology Research Center of Microorganisms and Biocatalysis, Hefei, 230601, China.

^4^ AHU Green Industry Innovation Research Institute, Hefei, 230088, China.

**SUPPORTING INFORMATION**

**Fig. S1** SDS-PAGE analysis of *B. subtilis* strains containing various promoters in shake flask fermentation.

**Fig. S2** SDS-PAGE analysis of WBZ-VY-B-R1 fermentation supernatant in a 3-L fermenter.

**Fig. S3** Growth curve of WBZ-VY-B-R1 cultured in a 3-L fermenter.

**Table S1** Nucleotide sequence of promoters.

**Table S2** Optimized RBS sequence information.

**Table S3** Primers used in this study.


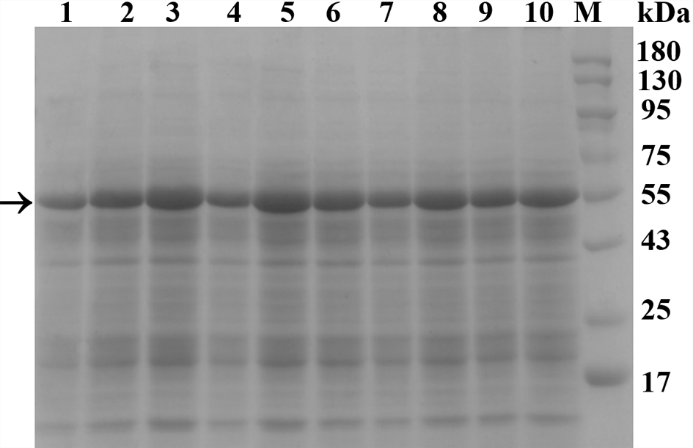


**Fig. S1** SDS-PAGE analysis of *B. subtilis* strains containing various promoters in shake flask fermentation.

The arrow indicates the band corresponding to AmyZ1 (~55 kDa). Lanes 1-10: supernatant samples from WBZ-Y, WBZ-YY, WBZ-VY, WBZ-SY, WBZ-YV, WBZ-VV, WBZ-SV, WBZ-YS, WBZ-VS, and WBZ-SS, respectively. Lane M: protein molecular weight markers.


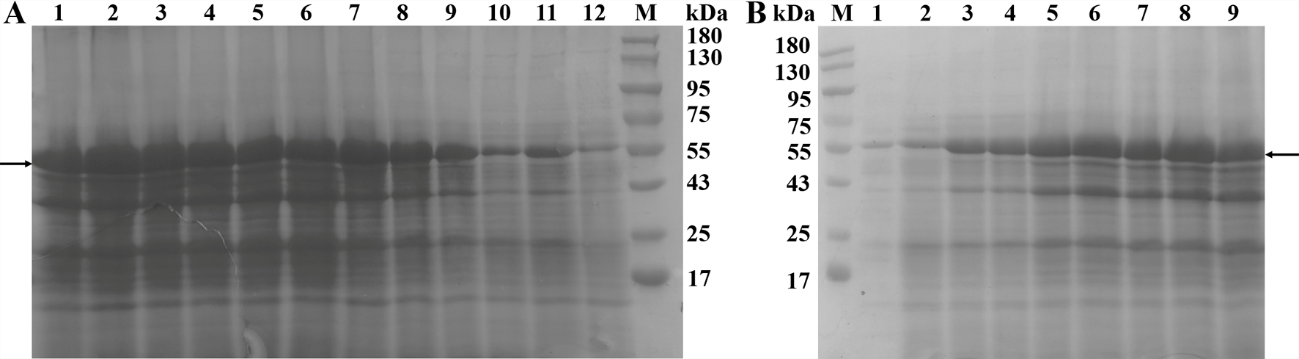


**Fig. S2** SDS-PAGE analysis of WBZ-VY-B-R1 fermentation supernatant in a 3-L fermenter.

(A) Lanes 1-3: supernatant samples at 64, 68, and 72 h at a 3:1 ratio, respectively. Lanes 4-6: supernatant samples at 64, 68, and 72 h at a 1:1 ratio, respectively. Lanes 7-9: supernatant samples at 60, 64, and 68 h at a 5:1 ratio, respectively. Lanes 10-12: supernatant samples at 52, 56, and 60 h at a 7:1 ratio, respectively. (B) Lanes 1-9: supernatant samples at 8, 24, 32, 40, 48, 56, 64, 68, and 72 h, respectively. The arrow indicates the band corresponding to AmyZ1 (~55 kDa). Lane M: protein molecular weight markers.


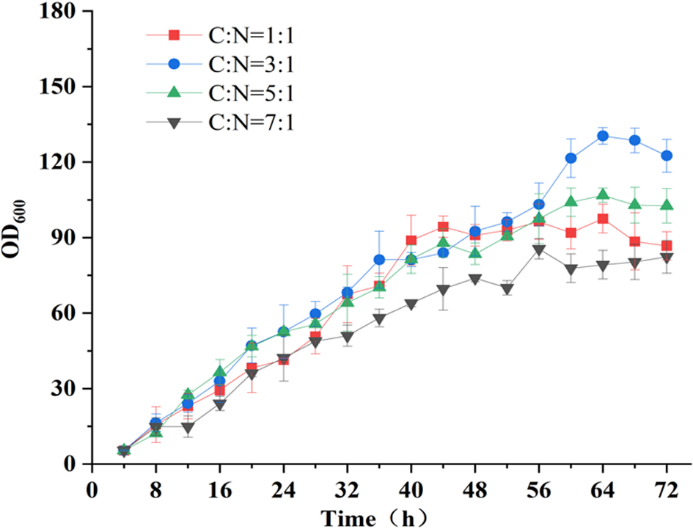


**Fig. S3** Growth curve of WBZ-VY-B-R1 cultured in a 3-L fermenter.

**Table S1** Nucleotide sequence of promoters.

| **Promoters** | **Nucleotide sequences** |
| --- | --- |
| P*_spoVG_* | TGCGGAAGTAAACGAAGTGTACGGACAATATTTTGACACTCACAAACCGGCGAGATCTTGTGTTGAAGTCGCGAGACTCCCGAAGGATGCGTTAGTCGAGATCGAAGTTATTGCACTGGTGAAATAATAAGAAAAGTGATTCTGGGAGAGCCGGGATCACTTTTTTATTTACCTTATGCCCGAAATGAAAGCTTTATGACCTAATTGTGTAACTATATCCTATTTTTTCAAAAAATATTTTAAAAACGAGCAGGATTTCAGAAAAAATCGTGGAATTGATACACTA |
| P*_veg_* | AGGAGTTCTGAGAATTGGTATGCCTTATAAGTCCAATTAACAGTTGAAAACCTGCATAGGAGAGCTATGCGGGTTTTTTATTTTACATAATGATACATAATTTACCGAAACTTGCGGAACATAATTGAGGAATCATAGAATTTTGTCAAAATAATTTTATTGACAACGTCTTATTAACGTTGATATAATTTAAATTTTATTTGACAAAAATGGGCTCGTGTTGTACAATAAATGTAGTGAGGTGGATGCAATG |
| P*_ylb_* | ACTTCTCAAAGATCCCATGTGCTTAAAATTAAAGTTTAAATATTTGGATTTTTTAAATAAAGCGTTTACAATATATGTAGAAACAACAAAGGGGGAGATTTGT |
| P*_HpaII_* | GATCTTCTCAAAAAATACTACCTGTCCCTTGCTGATTTTTAAACGAGCACGAGAGCAAAACCCCCCTTTGCTGAGGTGGCAGAGGGCAGGTTTTTTTGTTTCTTTTTTCTCGTAAAAAAAAGAAAGGTCTTAAAGGTTTTATGGTTTTGGATCGGCCACTGCCGACAGCCTCGCAGAGCACACACTTTATGAATATAAAGTATAGTGTGTTATACTTTACTTGGAAGTGGTTG |
| P*_hag_* | CGGTTGAAGGGGATCAAGTGAAGCTTGGAATTGACGCCCCAAAGCATATTGATATTCACAGGAAAGAAATTTACTTGACCATTCAGGAAGAAAATAACCGTGCAGCAGCGTTATCCAGCGATGTGATCTCCGCATTATCCTCACAAAAAAAGTGAGGATTTTTTTATTTTTGTATTAACAAAATCAGAGACAATCCGATATTAATGATGTAGC |

**Table S2** Optimized RBS sequence information.

| **RBS** | **Sequence** | **AmyZ1 activity (U/mL)** | **Translation initial rate (au)** |
| --- | --- | --- | --- |
| RBS0 | AAAGGAGGAAGGATCA | 4199.1 | 1714406.967 |
| RBS1 | AAAGGAGGTTTTGGAA | 4824.2 | 1868068.013 |
| RBS2 | AAAGGAGGTGTTAGAA | 2639.1 | 1760246.408 |
| RBS3 | AAAGGAGGTTTTACAA | 4251.0 | 1705887.779 |
| RBS4 | AAAGGAGGTTTGGGAA | 2644.5 | 1695631.205 |
| RBS5 | AAAGGAGGTTATAGAA | 3287.7 | 1651200.015 |
| RBS6 | AAAGGAGGTTTGAGAA | 2575.4 | 1549676.089 |
| RBS7 | AAAGGAGGTTAGGGAA | 3603.4 | 1539111.619 |
| RBS8 | AAAGGAGGTTATACAA | 3210.9 | 1514649.072 |
| RBS9 | AAAGGAGGTGTGAGTA | 2999.2 | 1470916.815 |
| RBS10 | AAAGGAGGTGAGAGTA | 2255.4 | 1453674.735 |

**Table S3** Primers used in this study.

| **Primer** | **Sequence (5'-3')** |
| --- | --- |
| F1 | CTGCAGATTATAGGTAAGAGAGGAAT |
| R1 | CATAGATGAATCCGAACCTCATTAC |
| PS-F | GTAATGAGGTTCGGATTCATCTATGGGGGTACCTGCGGAAGTAAAC |
| PS-R | TCCTCTCTTACCTATAATCTGCAGCTATATAAAAGCATTAGTGTA |
| PV-F | GTAATGAGGTTCGGATTCATCTATGAGGAGTTCTGAGAATTGGTATGC |
| PV-R | TCCTCTCTTACCTATAATCTGCAGCATTGCATCCACCTCACTACATTTATTG |
| PY-F | GTAATGAGGTTCGGATTCATCTATGACTTCTCAAAGATCCCATGTG |
| PY-R | TCCTCTCTTACCTATAATCTGCAGACAAATCTCCCCCTTTGTTGTTTC |
| PH-F | GTAATGAGGTTCGGATTCATCTATGGATCTTCTCAAAAAATACTACCTGTC |
| PH-R | TCCTCTCTTACCTATAATCTGCAGCAACCACTTCCAAGTAAAGTATA |
| PG-F | GTAATGAGGTTCGGATTCATCTATGCGGTTGAAGGGGATCAAGTGAAGC |
| PG-R | TCCTCTCTTACCTATAATCTGCAGGCTACATCATTAATATCGGATTGTCTC |
| F2 | ACTTCTCAAAGATCCCATGTGCTT |
| R2 | CATAGATGAATCCGAACCTCATTAC |
| PYY-F | GAGGTTCGGATTCATCTATGACTTCTCAAAGATCCCATGTG |
| PYY-R | AAGCACATGGGATCTTTGAGAAGTACAAATCTCCCCCTTTG |
| PVY-F | GAGGTTCGGATTCATCTATGAGGAGTTCTGAGAATTGG |
| PVY-R | AAGCACATGGGATCTTTGAGAAGTCATTGCATCCACCTCACTA |
| PGY-F | GAGGTTCGGATTCATCTATGGGGGTACCTGCGGAAGTA |
| PGY-R | AAGCACATGGGATCTTTGAGAAGTCTATATAAAAGCATTAGTGTA |
| F3 | AGGAGTTCTGAGAATTGGTAT |
| R3 | CATAGATGAATCCGAACCTCATTAC |
| PYV-F | GGTTCGGATTCATCTATGACTTCTCAAAGATCCCATG |
| PYV-R | ATACCAATTCTCAGAACTCCTACAAATCTCCCCCTTTG |
| PVV-F | GGTTCGGATTCATCTATGAGGAGTTCTGAGAATTGG |
| PVV-R | ATACCAATTCTCAGAACTCCTCATTGCATCCACCTCACTA |
| PGV-F | GGTTCGGATTCATCTATGGGGGTACCTGCGGAAGTA |
| PGV-R | ATACCAATTCTCAGAACTCCTCTATATAAAAGCATTAGTGTA |
| F4 | GGGGTACCTGCGGAAGTAAACGAAG |
| R4 | CATAGATGAATCCGAACCTCATTAC |
| PYS-F | GGTTCGGATTCATCTATGACTTCTCAAAGATCCCATG |
| PYS-R | CTTCGTTTACTTCCGCAGGTACCCCACAAATCTCCCCCTTTG |
| PVS-F | GGTTCGGATTCATCTATGAGGAGTTCTGAGAATTGG |
| PVS-R | CTTCGTTTACTTCCGCAGGTACCCCCATTGCATCCACCTCACTA |
| PGS-F | GGTTCGGATTCATCTATGGGGGTACCTGCGGAAGTA |

**Table S3** (continued)

| **Primer** | **Sequence (5'-3')** |
| --- | --- |
| PGS-R | CTTCGTTTACTTCCGCAGGTACCCCCTATATAAAAGCATTAGTGTA |
| F5 | GGATCCATGGCAAGCAAGAATGGGACCATGATG |
| R5 | TGATCCTTCCTCCTTTAATTGGGCTAATAGTTGA |
| F6 | GTTTAATGGTTCCGCAACAGATCCAAGGCGCATCTTCGGGATC |
| R6 | CCATTTTTTCATTGATCCTTCCTCCTTTAATTGGGCTAATAG |
| R1-F | CTATTAGCCCAATTAAAGGAGGTTTTGGAAATGAAAAAATGG |
| R1-R | GCAAGAAACAGGCCTGCCATCCATTTTTTCATTTCCAAAACCTCCTTT |
| R2-F | CTATTAGCCCAATTAAAGGAGGTGTTAGAAATGAAAAAATGG |
| R2-R | GCAAGAAACAGGCCTGCCATCCATTTTTTCATTTCCAAAACCTCCTTT |
| R3-F | CTATTAGCCCAATTAAAGGAGGTTTTACAAATGAAAAAATGG |
| R3-R | GCAAGAAACAGGCCTGCCATCCATTTTTTCATTTCCAAAACCTCCTTT |
| R4-F | CTATTAGCCCAATTAAAGGAGGTTTGGGAAATGAAAAAATGG |
| R4-R | GCAAGAAACAGGCCTGCCATCCATTTTTTCATTTCCAAAACCTCCTTT |
| R5-F | CTATTAGCCCAATTAAAGGAGGTTATAGAAATGAAAAAATGG |
| R5-R | GCAAGAAACAGGCCTGCCATCCATTTTTTCATTTCCAAAACCTCCTTT |
| R6-F | CTATTAGCCCAATTAAAGGAGGTTTGAGAAATGAAAAAATGG |
| R6-R | GCAAGAAACAGGCCTGCCATCCATTTTTTCATTTCCAAAACCTCCTTT |
| R7-F | CTATTAGCCCAATTAAAGGAGGTTAGGGAAATGAAAAAATGG |
| R7-R | GCAAGAAACAGGCCTGCCATCCATTTTTTCATTTCCAAAACCTCCTTT |
| R8-F | CTATTAGCCCAATTAAAGGAGGTTATACAAATGAAAAAATGG |
| R8-R | GCAAGAAACAGGCCTGCCATCCATTTTTTCATTTCCAAAACCTCCTTT |
| R9-F | CTATTAGCCCAATTAAAGGAGGTGTGAGTAATGAAAAAATGG |
| R9-R | GCAAGAAACAGGCCTGCCATCCATTTTTTCATTTCCAAAACCTCCTTT |
| R10-F | CTATTAGCCCAATTAAAGGAGGTGAGAGTAATGAAAAAATGG |
| R10-R | GCAAGAAACAGGCCTGCCATCCATTTTTTCATTTCCAAAACCTCCTTT |
| F7 | GGCAGAGTGACAGGTGGT |
| R7 | GGCAACTAAGATCAAGGGTT |
| F8 | CGACGCAGTCAAGCACATAAAGC |
| R8 | AGTTCTCCCACATCGTTCTTCC |
